# A phase 1, first-in-human, dose escalation study of JNJ-80038114, a PSMAxCD3 bispecific antibody, in participants with metastatic castration-resistant prostate cancer

**DOI:** 10.1007/s00280-025-04846-w

**Published:** 2026-01-06

**Authors:** Andrew Hudson, Anuradha Jayaram, Benjamin Garmezy, Nicholas A. Zorko, Kevin K. Zarrabi, Ligi Mathews, Brent Rupnow, Mengjie Li, Debopriya Ghosh, Karen Urtishak, Peter Francis, Sherry C. Wang, Edward Attiyeh, Johann de Bono

**Affiliations:** 1 The Christie NHS Foundation Trust, Manchester, UK; 2 UCLH Foundation Trust, London, UK; 3 Sarah Cannon Research Institute, Nashville, TN USA; 4 University of Minnesota Masonic Cancer Center, Minneapolis, MN USA; 5 Thomas Jefferson University Hospital, Philadelphia, PA USA; 6 Johnson & Johnson, 851 Duportail Road, Chesterbrook, PA 19087 USA; 7 Johnson & Johnson, Spring House, PA USA; 8 Johnson & Johnson, Raritan, NJ USA; 9 Royal Marsden NHS Foundation Trust, Surrey, UK

**Keywords:** JNJ-80038114, PSMA, Bispecific antibody, Metastatic castration-resistant prostate cancer, First-in-human

## Abstract

**Purpose:**

Prostate-specific membrane antigen (PSMA) has been identified as a therapeutic target for metastatic castration-resistant prostate cancer (mCRPC). The recent success of radioligands targeting PSMA spurred development of new PSMA-targeting agents including immunotherapy. JNJ-80038114 is a bispecific antibody that binds PSMA on tumor cells and CD3 on T cells to induce anti-tumor activity.

**Methods:**

This was a phase 1, open-label, multicenter study of JNJ-80038114 in participants with mCRPC and ≥ 1 prior systemic therapy. JNJ-80038114 was administered subcutaneously every 3 weeks (Q3W), starting at 0.1 mg. The primary endpoint was safety. Secondary endpoints included pharmacokinetics (PK), immunogenicity, and prostate-specific antigen (PSA).

**Results:**

At final analysis, 39 participants received 0.1–180 mg JNJ-80038114 across 11 dose-escalation cohorts for a median of 9.3 weeks (range, 0.1–31.1). The most common treatment-related adverse events (TRAEs; ≥20%) included cytokine release syndrome (CRS, 51.3%, all Grade 1–2), injection-site reactions (46.2%), and fatigue (30.8%). Related Grade ≥ 3 TRAEs occurred in 51.3% of participants; dose-limiting toxicities occurred in 3 (7.7%). Four participants (10.3%) developed clinically significant neuropathies. In 37 PK-evaluable participants, mean exposure (C_max_, AUC) increased with increasing doses. Anti-drug antibodies (ADA) were reported in 56.8% (21/37) participants. One participant had confirmed PSA decrease ≥ 50%. Three participants had radiological responses in the context of rapidly rising PSA. Following a review of data, the study was terminated.

**Conclusions:**

This first-in-human study of JNJ-80038114 was discontinued early due to its lack of preliminary clinical activity, neurologic toxicities, high rates of CRS, and the development of ADAs impacting PK.

**Clinicaltrial.Gov information:**

NCT05441501, Registered July 1, 2022.

## Introduction

Prostate-specific membrane antigen (PSMA) is a transmembrane glycoprotein with folate hydrolase activity [1]. PSMA is highly overexpressed in the vast majority of prostate adenocarcinomas [1], making it a potential therapeutic target for prostate cancer. While lutetitum vipivotide is to date the only PSMA-targeted treatment for metastatic castration-resistant prostate cancer (mCRPC) approved in the US and Europe, other candidate agents are currently being investigated, including radioligand therapies, antibody–drug conjugates, chimeric antigen receptor T cell (CAR T) therapy, and T cell engagers [2]. Immunotherapy approaches for prostate cancer have had limited success, likely due to immunologically ‘cold’ tumor microenvironments [3]. One of the recent approaches to overcome immune evasion involves T cell engagers or bispecific antibodies that simultaneously bind antigens on immune effector cells and tumor cells, thus directing activated T cells into the tumor [4].

PSMA as a potential therapeutic target for T cell redirection in prostate cancer was shown in a previous phase 1 study of JNJ-63898081, a first-generation T cell engager binding to PSMA-expressing cdose-limiting toxicities (DLTs) were observedells and CD3-expressing T cells, administered by intravenous or subcutaneous (SC) dosing in 39 participants with mCRPC [5]. In that study, in 4 participants, and Grade 1–2 cytokine release syndrome (CRS) was more frequently observed at higher intravenous and SC doses, but was manageable with a combination of SC dosing and/or step-up priming. No treatment-related deaths occurred. Transient declines in prostate-specific antigen (PSA) were recorded but no radiographic responses were observed. This study was terminated early based on emerging data showing a negative impact of anti-drug antibodies (ADAs) on JNJ-63898081 pharmacokinetics (PK).

JNJ-80038114 is a novel, fully human immunoglobulin G1 (IgG1)-derived bispecific monoclonal antibody targeting the CD3ε subunit of the T cell receptor and PSMA. JNJ-80038114 enables T cell-mediated cytotoxicity by recruiting CD3-expressing T cells to PSMA-expressing target cells. Furthermore, it was engineered such that the PSMA binding arm has very high affinity for antigen, allowing sustained target cell engagement while the CD3ε engaging arm has weak affinity, potentially mitigating adverse cytokine responses. Based on a previous study [5], the difference in affinities was thought to improve the efficacy and safety of the T cell engager. JNJ-80038114 has demonstrated specific cytotoxicity to PSMA-positive prostate cancer cells in vitro and in vivo. We report herein the first-in-human study of JNJ-80038114 in mCRPC (NCT05441501).

## Materials and methods

### Participants

Eligible participants were ≥ 18 years old, had mCRPC with confirmed adenocarcinoma of the prostate (small cell or neuroendocrine features were permitted), measurable/evaluable disease, and ≥ 1 prior line of systemic therapy for mCRPC (androgen receptor pathway inhibitor [ARPI] or chemotherapy). Additionally, participants must have had prior orchiectomy or ongoing androgen deprivation therapy (ADP) with a gonadotropin-releasing hormone analog that was required to continue throughout the study treatment phase, Eastern Cooperative Oncology Group (ECOG) performance score 0–1, and adequate organ function. Key exclusion criteria were a history of central nervous system or leptomeningeal involvement, any prior PSMA-targeting antibody, or prior T cell directing treatment within 90 days of study treatment initiation.

The study was conducted in accordance with the Declaration of Helsinki, Good Clinical Practice, and applicable regulatory requirements. The study protocol was approved by the Institutional Review Board/Independent Ethics Committee at each study site, and written informed consent was obtained from all participants.

### Study design

This was a first-in-human, open-label, multicenter, phase 1 study of JNJ-80038114 in participants with mCRPC, who had at least one prior line of therapy, designed with two parts: dose escalation and dose expansion. The primary objectives were to assess safety and to determine the recommended phase 2 dose and optimal dosing schedules. Secondary objectives were the assessment of PK/pharmacodynamics (PD), immunogenicity, and anti-tumor activity.

JNJ-80038114 was administered at a SC starting dose of 0.1 mg every 3 weeks (Q3W) based on the minimum anticipated biologic effect level [6], and subsequent dose levels and step-up dosing schedules were determined by a study evaluation team (SET) based on the totality of the data, including emerging safety and PK data. Prior to drug administration, all participants were required to receive antihistamine (diphenhydramine), antipyretic (acetaminophen), and glucocorticoid (dexamethasone) therapies. Participants were hospitalized for ≥ 36 h after the first full treatment and the first step-up dose to monitor safety. All other study treatment administrations were given as outpatient unless hospitalization for subsequent doses was considered necessary by the study investigator.

### Study assessments

Safety assessments included adverse events (AEs) and DLTs. CRS and associated neurologic toxicity events (i.e., immune effector cell-associated neurotoxicity syndrome [ICANS]) were graded according to the American Society for Transplantation and Cellular Therapy guidelines [7]. All other AEs were graded according to the National Cancer Institute Common Terminology Criteria for Adverse Events version 5.0.

Clinical activity according to Prostate Cancer Clinical Trials Working Group 3 (PCWG3) criteria was assessed by the investigator using computed tomography scans or magnetic resonance imaging, and whole-body bone scans (^99m^Tc). Additional evaluation of efficacy included serum PSA. Blood samples were collected to characterize PK and ADAs to JNJ-80038114.

### Statistical analysis

No formal statistical hypothesis testing was planned. Dose escalation was guided by the Bayesian optimal interval design [8] with a target DLT rate of *≤* 30%. Safety and efficacy analyses included all participants who received ≥ 1 JNJ-80038114 dose and were summarized using descriptive statistics.

## Results

### Participants

Thirty-nine participants were treated at six centers in the USA and United Kingdom from November 2022 to March 2024. The median (range) age was 67.0 (50–84) years, and 33 participants had ECOG status of 1 (6 had ECOG 0). Nine participants (23.1%) had metastatic disease to the lung, liver, adrenal glands, or central nervous system. Median PSA at baseline was 189 ug/L (range: 5–3074). All participants had prior cancer-related therapy including ADP and an androgen receptor-targeted therapy or chemotherapy. Thirty-seven participants (94.9%) received prior ARPI, 34 (87.2%) had received taxanes, 12 (30.8%) had received prior radioligand therapy, and 6 (15.4%) had received prior poly-ADP ribose polymerase inhibitors. The median treatment duration across the 11 dose escalation cohorts was 9.3 (range, 0.1–31.1) weeks. Five participants died during the study due to progressive disease. When the study was terminated, treatment was discontinued for all remaining participants. Intra-participant dose escalation occurred in 14/39 participants, as permitted per protocol. Step-up dosing was conducted for all doses above 100 mg to mitigate CRS.

### Safety

All 39 participants reported all-causality treatment-emergent adverse events (TEAEs) (Table 1). The most frequent treatment-related adverse events (TRAEs) (≥ 20%) were CRS (51.3% overall, including 35.9% Grade 1 and 15.4% Grade 2), ifatigue (48.7%), injection-site reactions (ISRs, 46.2%), lymphopenia (43.6%), diarrhea (35.9%), nausea (25.6%), and increased aspartate aminotransferase (25.6%). The most frequently reported symptoms of CRS (> 10%) were pyrexia (28.2%) and hypotension (10.3%). The majority of CRS events began 1–3 days after study-drug administration and resolved within 2 days (35% received tocilizumab). Though a clear dose response could not be established due to small participant numbers, CRS was numerically more frequent at higher doses of JNJ-80038114.

**Table 1:** Treatment-emergent adverse events

*n* (%)	Total (*N* = 39)
Any TEAEs	39 (100)
Related to study drug	37 (94.9)
Grade 3–4	29 (74.4)
Related to study drug	20 (51.3)
Serious AEs	20 (51.3)
Related to study drug	12 (30.8)
AEs leading to discontinuation	6 (15.4)
Related to study drug	3 (7.7)
Most common TRAEs (> 10%)	
Cytokine release syndrome	20 (51.3)
Fatigue	19 (48.7)
Injection site reaction	18 (46.2)
Lymphopenia	17 (43.6)
Diarrhea	14 (35.9)
Decreased appetite	12 (30.8)
Nausea	12 (30.8)
Anemia	22 (28.2)
Aspartate aminotransferase increased	10 (25.6)
Constipation	10 (25.6)
Dysgeusia	8 (20.5)
Rash, maculo-papular	8 (20.5)
Arthralgia	7 (17.9)
Headache	7 (17.9)
Pyrexia	7 (17.9)
Vomiting	7 (17.9)
Alanine aminotransferase increased	6 (15.4)
Hypokalemia	6 (15.4)
Rash, macular	6 (15.4)
Thrombocytopenia	6 (15.4)
Dizziness	5 (12.8)
Dry mouth	5 (12.8)
Muscle weakness	5 (12.8)
Neutropenia	5 (12.8)
Pain in extremity	5 (12.8)
Peripheral edema	5 (12.8)
Pruritis	5 (12.8)
Hypophosphatemia	4 (10.3)
Oropharyngeal pain	4 (10.3)
Paresthesia	4 (10.3)
Rash	4 (10.3)
Weight decreased	4 (10.3)
Most common Grade ≥ 3 TRAEs (> 5%)	
Lymphopenia	13 (33.3)
Anemia	7 (17.9)
Aspartate aminotransferase increased	3 (7.7)
Fatigue	3 (7.7)
Neutropenia	3 (7.7)
Acute kidney injury	2 (5.1)
Arthralgia	2 (5.1)
Thrombocytopenia	2 (5.1)

TEAE, treatment-emergent adverse event, TRAE, treatment-related adverse event

Grade ≥ 3 TRAEs occurred in 51.3% of participants, most commonly (> 10% of participants) lymphopenia (33.3%). DLTs occurred in 3 (7.7%) participants, which included reversible multiple cranial nerve polyneuropathy (Grade 2, onset on study Day 6, after only one 100 mg dose), peripheral sensory neuropathy (Grade 3, onset on study Day 17, after receiving 30 mg on Day 1 and 100 mg dose on Day 9) in a patient with a history of peripheral neuropathy, and the third participant reported hepatitis (Grade 2) and pancreatitis (Grade 3) after the 30 mg dose (onset on study Day). Two participants developed peripheral neuropathies at the 180 mg dose level: one had Grade 1 CRS followed by an ascending peripheral neuropathy up to T10 and the other had Grade 2 CRS followed by loss of vibration sensation and proprioception of both legs. At the time of database lock, the DLTs of hepatitis/pancreatitis and peripheral sensory neuropathy were reported as recovered/resolving and the DLT of cranial nerve disorder as not resolved.

Serious all-causality TEAEs were reported in 20 participants (51.3%, Table 1), all of which were limited to 1 or 2 participants except for CRS (*n* = 5; 12.8%, all Grade < 3). TRAEs leading to treatment discontinuation were reported in 3 participants. Five participants died during the study due to disease progression, and one additional participant died after database lock (the investigator reported that both disease progression and study drug toxicity may have contributed).

### Pharmacokinetics and immunogenicity

PK data from 37 participants who received JNJ-80038114 and had appropriate blood collections after SC injections of JNJ-80038114 were available. Following the first dose of JNJ-80038114 ranging from 1.0 to 180 mg Q3W, both maximum observed concentration (C_max_) and area under the concentration vs. time curve from day 0–21 (AUC_0 − 21d_) increased with increasing dose. Median time to reach maximum observed concentration (t_max_) was between 71 and 154 h. Steady state was achieved following the fourth dose.

Treatment-emergent ADAs to JNJ-80038114 were detected in 21 (56.8%) participants **(**Table 2**)**, of which 7 (33.3%) showed non-detectable JNJ-80038114 pre-dose serum concentration at the time of peak ADA titer, although no assays to detect neutralizing antibodies were performed. Analysis of these 21 ADA-positive participants suggests those with higher titers of ADA generally showed lower serum concentrations of study drug. ADA positive status was likely associated with decline of systemic exposure to JNJ-80038114.

**Table 2:** Anti-drug antibodies to JNJ-80038114^a^

	Q3W SC (Without Step Up)							Q3W SC (With Step Up)				Total (*N* = 37)
*n* (%)	0.1 mg (*n* = 1)	0.3 mg (*n* = 2)	1 mg (*n* = 4)	3 mg (*n* = 4)	10 mg (*n* = 3)	30 mg (*n* = 7)	100 mg (*n* = 4)	30 then 100 mg (*n* = 1)	10 and 30, then 60 mg (*n* = 4)	10 and 30, then 100 mg (*n* = 3)	10 and 30, then 180 mg (*n* = 4)	
Positive for ADAs	0	2 (100)	2 (50.0)	2 (50.0)	3 (100)	3 (42.9)	3 (75.0)	0	2 (50.0)	2 (66.6)	2 (50.0)	21 (56.8)
Negative for ADAs	1 (100)	0	2 (50.0)	2 (50.0)	0	4 (57.1)	1 (25.0)	1 (100)	2 (50.0)	1 (33.3)	2 (50.0)	16 (43.2)

^a^In participants with ≥ 1 appropriate samples obtained after JNJ-80038114 administration. ADAs, anti-drug antibodies; Q3W, every 3 weeks; SC, subcutaneous

### Preliminary anti-tumor activity

Four (10.3%) of 39 participants had a ≥ 50% PSA decrease at any time, including 1 participant who had a confirmed ≥ 50% PSA decrease with JNJ-80038114 10 mg Q3W at Week 6. Three participants with measurable disease had partial response per PCWG3 criteria, but all had rapidly rising PSA.

### Early study termination

Following the SET’s review of the benefit-risk profile of JNJ-80038114 based on available safety, efficacy, and PK data, the sponsor made the decision in March 2024 to discontinue the dose escalation phase and not initiate the dose expansion phase.

## Discussion

The most frequently reported TRAEs (> 40%) were CRS, fatigue, SRs, and lymphopenia. More than half of participants developed ADAs. Due to the minimal signs of anti-tumor activity (10.3% of participants had transient PSA decreases, including only 1 confirmed PSA50 response; 3 participants had partial response by PCWG3 criteria in the context of rapidly rising PSA) and high incidence of CRS, the study was terminated.

The development of T cell engagers for the treatment of patients with mCPRC was prompted by their success in hematological malignancies. Multiple clinical programs with T cell engagers are ongoing in solid tumors, including mCPRC [9, 10]. The T cell redirection approach is viewed as particularly promising in the treatment of prostate cancer, which has an immunologically ‘cold’ tumor microenvironment [3]. However, several clinical development programs of T cell engagers and bispecific antibodies in mCRPC, including acapatamab [9], JNJ-63898081 [5], and AMG340 [10] were recently terminated due to substantial toxicity and modest anti-tumor activity.

In this study, ADAs were detected in 56.8% of evaluable participants with appropriate samples. Analysis of those ADA-positive participants suggests that participants with higher titers of ADA generally showed lower serum concentrations of study drug. As the PK data may have been affected by early discontinuation and undetectable concentrations at low doses, no firm conclusions can be drawn on the effect of ADA on PK. The development of ADAs is one of the key challenges of bispecific antibodies and T cell engagers due to their abnormal structures and bioengineered sequences [11]. While this study used SC administration of JNJ-80038114 and reported high incidence of ADAs, results from other studies have been mixed with regards to associations between the route of administration (intravenous versus SC) and the incidence of ADAs [12].

The incidence of CRS in this study (51.3%) was broadly similar to those previously reported with AMG340 (52%), HPN424 (63%), and JNJ-63898081 (67%) and lower than 86% with CC1 or 97–98% with acapatamab [5, 9, 10, 13]. In our study, CRS was reported as a serious TRAE in 12.8% of participants. None of the CRS events led to study discontinuation. High rates of CRS have been observed with T cell engagers targeting PSMA and STEAP1, in contrast to very low CRS rates reported for pasritamig, a T cell engager targeting KLK2, which may support a relationship with limited normal tissue expression and CRS [14, 15]. DLTs were reported in three participants who received JNJ-80038114 at 30 mg and 100 mg. The neuropathies observed in this trial, which included reversible polyneuropathy of multiple cranial nerves, have also not been observed with T cell engagers targeting other targets and suggest that PSMA-targeted T cell engagers may drive T cells to neurons directly or indirectly perhaps due to PSMA expression on nerves.

Nonetheless, PSMA remains a potential therapeutic target for T-cell redirection for the treatment of mCRPC. Ongoing clinical studies explore the safety and anti-tumor activity of other PSMAxCD3 targeted agents, including CCW702 [16] and JANX-007 [17]. Additional T-cell redirection approaches include a bi-ligand antibody drug conjugate CBP-1018 [18] targeting PSMA and FRα, and bispecific antibodies directed against PSMA/CD28 (nezastomig) [19] and PSMA/Vδ2 chain (LAVA-1207) [20].

To mitigate toxicity and maximize efficacy, T-cell engagers are being engineered with new technologies. Early studies are underway for T-cell engagers that have varying affinities for their targets, particularly CD3, to improve potency (affinity tuning). Although potentially useful for reducing the frequency and severity of CRS, it is possible that the weaker CD3 affinity of JNJ-80038114 contributed to its poor anti-tumor activity. Prodrug-like T-cell engagers are also being developed that have temporarily inactive or blocked binding sites. These agents circulate in an inert form and are activated by the tumor microenvironment (masking), thereby improving their safety profiles [21].

Although this first-in-human study demonstrated a manageable safety profile of JNJ-80038114, the lack of anti-tumor activity and the immunogenicity results do not support further development for mCRPC. These data contribute to the emerging clinical experience with T-cell engagers and bispecific antibodies in prostate cancer. Additional exploration of PSMA/CD3 bispecific antibodies, including optimal dosage, half-life, and delivery is warranted.

## Data Availability

The data sharing policy of Johnson & Johnson is available at https://innovativemedicine.jnj.com/our-innovation/clinical-trials/transparency. Although these data are not currently publicly available for sharing, requests for sharing can be sent to the Corresponding Author and will be evaluated on an individual basis.
